# Feasibility of flow cytometric analysis of restricted light chain in endoscopic biopsy specimens from patients with gastrointestinal tract B cell lymphoma: a pilot study

**DOI:** 10.1186/s13104-019-4578-4

**Published:** 2019-09-11

**Authors:** Katsunori Matsueda, Masaya Iwamuro, Takahide Takahashi, Sizuma Omote, Kenji Nishida, Takehiro Tanaka, Daisuke Ennishi, Fumio Otsuka, Tadashi Yoshino, Hiroyuki Okada

**Affiliations:** 1 Department of Gastroenterology and Hepatology, Okayama University Graduate School of Medicine, Dentistry and Pharmaceutical Sciences, 2-5-1 Shikata-cho, Kita-Ku, Okayama, 700-8558 Japan; 2 Division of Medical Support, Okayama University Hospital, Okayama, 700-8558 Japan; 3 Department of Pathology, Okayama University Graduate School of Medicine, Dentistry and Pharmaceutical Sciences, Okayama, 700-8558 Japan; 4 Department of Hematology and Oncology, Okayama University Graduate School of Medicine, Dentistry and Pharmaceutical Sciences, Okayama, 700-8558 Japan; 5 Department of General Medicine, Okayama University Graduate School of Medicine, Dentistry and Pharmaceutical Sciences, Okayama, 700-8558 Japan

**Keywords:** Flow cytometric analysis, Endoscopic biopsy specimens, Gastrointestinal tract B-cell lymphoma, Restricted light chain, Light chain expression

## Abstract

**Objective:**

Gastrointestinal tract lymphomas are currently detected more frequently due to advances in endoscopic technology. The aim of this study was to assess the feasibility of flow cytometric analysis of restricted light chain in endoscopic biopsy specimens for the diagnosis of gastrointestinal tract B-cell lymphoma. We prepared viable cell suspensions from unfixed specimens obtained from 10 consecutive patients who had a previous histological diagnosis of gastrointestinal tract B-cell lymphoma. We performed immunophenotypic studies with multi-color flow cytometry and assessed clonality through examination of immunoglobulin light chain expression exclusively in a population identified by anti-CD45 or CD20 antibodies.

**Results:**

We could perform light chain expression analysis with 2 endoscopic biopsy specimens from all 10 patients with gastrointestinal tract B-cell lymphoma. We conclude that flow cytometric analysis of endoscopic biopsy specimens is feasible and thus likely useful for the diagnosis of gastrointestinal tract B-cell lymphoma in clinical settings.

*Trial registration* UMIN Clinical Trials Registry, UMIN000027730. Registered 12 June 2017

## Introduction

Flow cytometric analysis of the lymph nodes, peripheral blood, and bone marrow is widely used for immunophenotyping of leukemia, lymphoma, myeloma, and myelodysplastic syndrome [1–3]. Although the diagnosis and classification of non-Hodgkin lymphomas are primarily made by histological examination [4], detection of clonality by flow cytometric analysis reinforces accurate diagnosis of this disease entity [3, 5].

The gastrointestinal tract is the most common site for extranodal B-cell lymphoma (BCL), which includes marginal zone BCL, follicular lymphoma (FL), and other subsets of BCL [6, 7]. Since almost all types of BCL show restricted light chain expression, flow cytometric analysis can theoretically be used to identify B-cell clonality by analyzing κ and λ ratios in a B-cell population [8]. Flow cytometric analysis of endoscopic biopsy specimens holds several potential advantages for the diagnosis of BCL. First, the number of neoplastic cells is sometimes small in biopsy specimens [9]. Although histological evaluation may be hampered owing to large amounts of normal lymphocytes in the background that obscures the lymphoma cells [10, 11], flow cytometric analysis is able to detect a few neoplastic cells even in a small sample [12]. Second, flow cytometric analysis requires only several hours to obtain the results, whereas histological analysis with immunostaining generally takes days to weeks. Early and accurate diagnosis of gastrointestinal tract BCLs by endoscopic biopsy specimens is of clinical importance to determine an appropriate treatment strategy [13, 14].

However, flow cytometric analysis of endoscopic biopsy specimens has not been conducted in routine clinical practice for the diagnosis of gastrointestinal tract BCL. The aim of this study is to prospectively assess the feasibility of flow cytometric analysis of restricted light chain in endoscopic biopsy specimens for the diagnosis of gastrointestinal tract BCL.

## Main text

### Methods

#### Patients

We performed flow cytometric analysis on ten patients with gastrointestinal tract BCLs between November 2017 and March 2018 at Okayama University Hospital and the results were analyzed prospectively. These patients had been diagnosed with lymphoma prior to flow cytometry (FCM) examination based on the histological examination of specimens obtained by endoscopic biopsy. Flow cytometric analysis was performed during the follow-up period.

#### Pathological analysis

We took one biopsy specimen from the lymphoma lesion using disposable biopsy forceps (EndoJaw™, FB-230K, Olympus, Tokyo, Japan) for conventional pathological analysis. The biopsy specimens were fixed in formalin and stained with Hematoxylin–Eosin. Immunostaining was also performed using a panel of monoclonal antibodies. Histological subtypes of lymphoma were defined according to the World Health Organization classification [4].

#### Sample preparation for flow cytometric analysis

Two additional specimens were taken from the same lesion of each patient and were used for flow cytometric analysis to analyze the expression of light chains and B-cell antigens including CD20 or CD19. Two endoscopically harvested specimens were put into a 15 mL conical centrifuge tube with 10 mL of normal saline solution. The saline solution containing specimens was decanted into a 100-mm petri dish (Dish A). Subsequently, we dissected the specimens into the finest pieces possible using a scalpel blade and tweezers. A wire mesh tea strainer, which had been sterilized by autoclaving prior to use, was placed in a new 100-mm petri dish (Dish B). The specimen pieces were then placed on a wire mesh tea strainer and the pieces were crushed using the rubber portion of the plunger of a 10 mL injection syringe. Fibrous residues left on the wire mesh tea strainer were removed with tweezers. We suck up the saline solution, filtered with the wire mesh tea strainer, and poured into the 100-mm petri dish (Dish B). Dish A was washed with a fresh saline solution, filtered through the wire mesh tea strainer, and collected into Dish B. The solution in dish B was transferred into a conical tube and centrifuged at 1500 rpm for 5 min at room temperature (approximately 15°). We removed the supernatant and suspended the sample in approximately 5 mL of saline solution.

#### Flow cytometric analysis

Samples were centrifuged at 360*g* for 5 min at room temperature immediately before flow cytometric analysis. After centrifugation, we removed the supernatant and resuspended with 500 μL of saline solution. The solution was used for flow cytometric analysis. The monoclonal antibodies for CD45 (J33; Beckman Coulter), CD19 (J3-119; Beckman Coulter), CD20 (B-Ly1; Dako), CD10 (ALB1; Beckman Coulter), CD3 (UCHT1; Beckman Coulter), and CD5 (BL1a; Beckman Coulter) and polyclonal antibodies for surface membranes κ (Polyclonal F(ab’)2; Dako) and λ (Polyclonal F(ab’)2; Dako) were used in multi-color FCM. The immunostained cells were analyzed by FACScan (Navios flow cytometer, Beckman Coulter, Brea, CA, USA), using Kaluza analysis software version 1.3. B-cell clonality was measured by κ/λ ratios, quantifying the κ and λ light chain expressions in a gated CD45 population (CD45+ cell population) or a gated CD20 population (CD20+ cell population). The primary outcome of this study was the feasibility of flow cytometric analysis to detect the expression of light chains in the 2 biopsy specimens, which were endoscopically taken from patients with gastrointestinal tract BCLs. We also examined patients’ clinicopathologic features based on their medical records, including the gender, age, site of primary gastrointestinal tract BCLs, histological subtype, chromosomal aberration, stage based on the Lugano International Conference classification, incidence of *Helicobacter pylori* infection, and treatment.

### Results

#### Patient characteristics

The characteristics of 10 patients, who were previously diagnosed with gastrointestinal tract BCL based on the histological examination of endoscopic biopsy specimens, are shown in Table 1. Three were males and 7 were females, and the median age was 68.5 years (range 50s–80s years). The histological subtypes were gastric extranodal marginal zone lymphoma of mucosa-associated lymphoid tissue (MALT lymphoma, N = 7) and duodenal FL, grade 1 (N = 3). All 10 patients had stage I disease.

**Table 1:** Patient characteristics

Case	Sex	Site of biopsy	Histologic diagnosis	Stage (Lugano classification)	Chromosomal aberration	HP	Eradication	CD45+ cell number	Antigen expression (%) in CD45+ population					κ/λ ratios
									CD19	CD20	CD10	Igκ	Igλ	
1	F	Stomach	MALT	I	None	No	Yes	2349	88.7	89.3	1.9	3.1	57.7	0.05
2	F	Stomach	MALT	I	*API2*-*MALT1*	No	No	1079	ND	72.9	1.6	61.6	6.8	9.06
3	F	Stomach	MALT	I	ND	Yes	Yes	5306	ND	82.5	5.6	39.6	26.8	1.48
4	M	Duodenum	FL	I	ND	No	No	27967	ND	89.4	48.5	84.3	1.1	76.64
5	F	Stomach	MALT	I	*API2*-*MALT1*	No	Yes	5186	ND	89.6	10.7	11.2	51.2	0.22
6	M	Stomach	MALT	I	None	No	No	3126	53.8	91.2	9.1	43.9	11.9	3.69
7	F	Duodenum	FL	I	ND	Yes	No	1760	50.9	49.5	37.5	36.7	1.5	24.47
8	F	Stomach	MALT	I	*API2*-*MALT1*	No	Yes	487	ND	71.2	6.9	45.4	11.4	3.98
9	M	Duodenum	FL	I	*IgH*-*BCL2*	No	No	14810	85.1	88.1	84.4	0.3	82.2	<0.01
10	F	Stomach	MALT	I	*API2*-*MALT1*	No	Yes	4756	ND	97.4	3.7	16.6	74.9	0.22

*MALT* MALT lymphoma, *FL* follicular lymphoma, *HP Helicobacter pylori*, *ND* not done

#### Pathological analysis

As described above, we endoscopically took three specimens from the lymphoma lesion with disposable biopsy forceps; one biopsy specimen was provided for conventional pathological analysis and the other two specimens were used for flow cytometric analysis. Pathology of the biopsy specimen were in accordance with the previous histological diagnosis in all cases.

#### Flow cytometric analysis

For flow cytometric analysis, 487 to 27,967 CD45+ cells (median: 3941 cells) were analyzed. In seven patients, more than 80% of the CD45+ cells expressed the B-cell antigen (CD20 or CD19). In MALT lymphomas, 1.6% to 10.7% of CD45+ cells were positive for CD10, whereas 37.5% to 84.4% of CD45+ cells were positive for CD10 in FLs. The light chain expression was successfully analyzed in all 10 patients (100%) by flow cytometric analysis.

#### Representative results of flow cytometric analysis

**Fig. 1:**
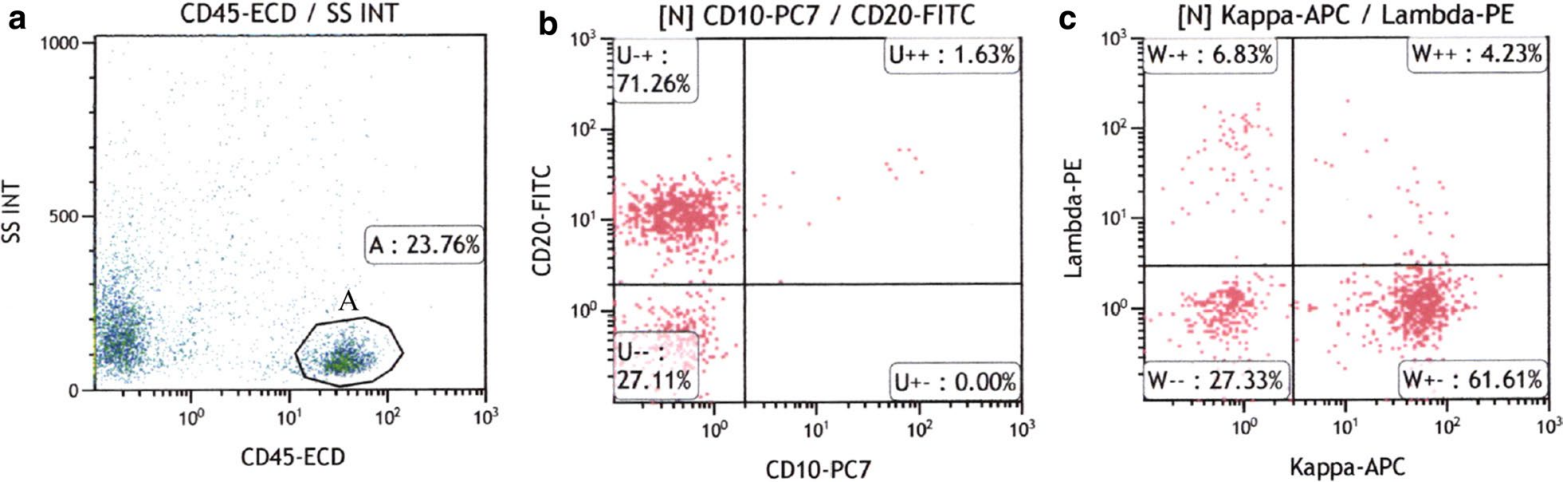
(Case 2) Flow cytometric analysis showing B-cell lymphomas. **a** Isolated cells (23.76%) (gate A) were positive for CD45. **b** Among the CD45+ cell populations, 72.89% of the cells were CD20+. **c** Light chain expression analysis showed dominant Igκ expression, resulting in κ/λ ratio of 9.06

**Fig. 2:**
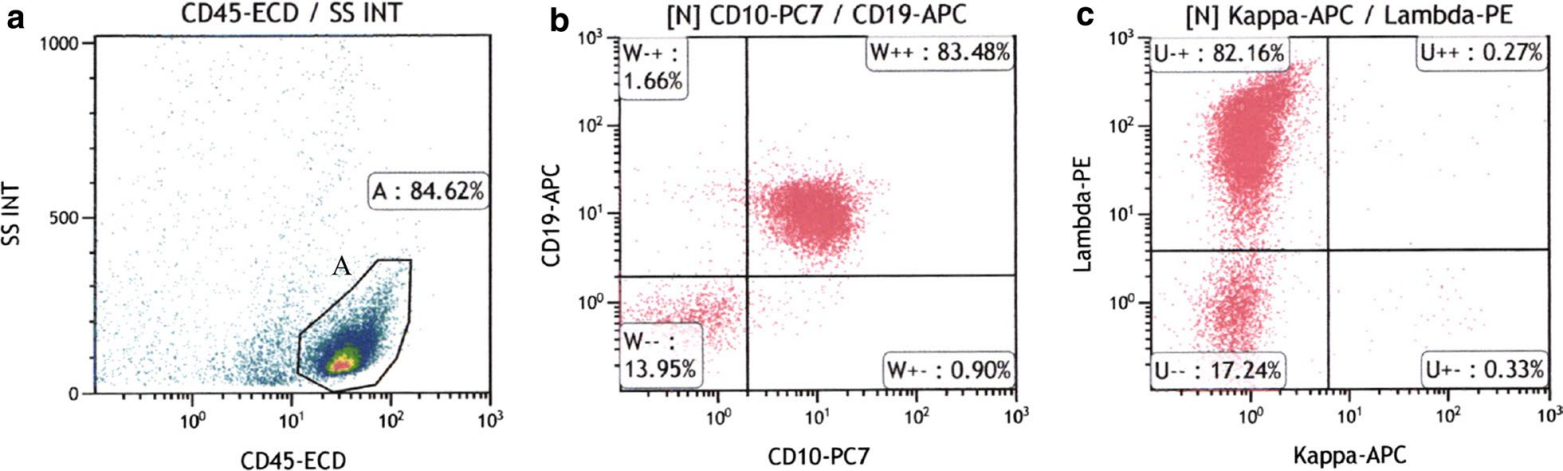
(Case 9) Flow cytometric analysis showing B-cell lymphomas. **a** Isolated cells (84.62%) (gate A) showed CD45 expression. **b** CD19+ and CD10+ cells were found in 85.14% and 84.38% of the CD45+ cells, respectively. **c** Dominant Igλ expression with κ/λ ratio of 0.0036 was observed

Figure 1 (gastric MALT lymphoma, case 2) and 2 (duodenal FL, case 9) show representative results of flow cytometric analysis, in which clonal B-cell populations were detectable. In Fig. 1, 23.76% of isolated cells were positive for CD45 (Fig. 1a, gate A). Among the CD 45+ cell populations, 72.89% of the cells were CD20+ (Fig. 1b). Light chain expression analysis showed dominant Igκ expression, resulting in κ/λ ratio of 9.06. These results were suggestive of clonal B-cells. In Fig. 2, 84.62% of the isolated cells displayed CD45 expression (Fig. 2a, gate A). CD19+ and CD10+ cells were found in 85.14% and 84.38% of the CD45+ cells, respectively (Fig. 2b). Dominant Igλ expression with κ/λ ratio of 0.0036 was observed, indicating the presence of clonal B-cells (Fig. 2c).

#### Better selection of clonal B-cells

Additional file 1: Fig. S1 (gastric MALT lymphoma, case 6) shows a case, in whom a subpopulation of cells with slightly decreased CD45 expression is evident. In Additional file 1: Fig. S1, 41.80% of the isolated cells showed CD45 expression (Additional file 1: Fig. S1A, gate A). Light chain expression analysis of gate A showed dominant Igκ expression with κ/λ ratio of 3.69 (Additional file 1: Fig. S1B). Analysis of subpopulation of cells with slightly decreased CD45 expression (Additional file 1: Fig. S1A, gate B) revealed more dominant Igκ expression with κ/λ ratio of 7.30 (Additional file 1: Fig. S1C), suggesting better selection of clonal B-cells. We performed the subpopulation analysis in the other 3 cases (cases 1, 5, and 7) and observed slightly more dominant light chain expression than in a CD45+ cell population (Additional file 2: Table S1).

### Discussion

In this study, we successfully performed light chain expression analysis with 2 endoscopic biopsy specimens from each of all 10 patients, although previous studies reported that an adequate number of cells in endoscopic biopsy specimens are essential for flow cytometric analysis [15, 16]. These CD45+ cells also allowed us to perform a limited but common immunophenotypic study, including the simultaneous analysis of light chain, CD20 or CD19, and CD10 antigen expression in each case.

Given that the previously published criteria for flow cytometric analysis of restricted light chain (normal range 0.5–3.0, any values outside this range are regarded as restricted light chain [3, 5, 17]) were applied to the endoscopic biopsy specimens in our study, 90% of the patients (9 of 10 patients) displayed restricted light chain expression, consistent with BCL, while one patient (case 3) did not. Several hypotheses are considered for the lack of restricted light chain expression. First, inadequate samples, owing to non-representative sampling or nonviable neoplastic cells, may lead to a false-negative result by flow cytometric analysis. Second, the existence of B cells with negative or dual positive expressions of κ and λ results in inconclusive restricted light chain expression analysis [18–20], despite the presence of neoplastic B-cells. In case 3, 8.1% of CD45+ cells were positive for both κ and λ and 25.5% of CD45+ cells were negative for κ and λ. Both percentages were higher than with any other cases. Therefore, the diversity of light chain expression among neoplastic B cells might have distorted analysis. Thirdly, the gating of B-cells, due to aberrant B-cell surface antigen expression by the lymphoma, was inappropriate, and therefore immunoglobulin light chain expression may not be detected.

CD45 is a marker of lymphocytes [21]. Hendrickx et al. investigated CD45 expression of normal B lymphocytes (N = 15), chronic lymphocytic leukemia (N = 22), mantle cell lymphoma (N = 2), and hairy cell leukemia (N = 6). The authors described that neoplastic B-cells in chronic lymphocytic leukemia and mantle cell lymphoma showed slightly decreased CD45 expression, whereas those in hairy cell leukemia showed slightly higher CD45 expression. Previously, we also reported a case of gastric MALT lymphoma, in which neoplastic B-cells were successfully separated by identifying a subpopulation of cells with slightly decreased CD45 expression [22]. In case 6, light chain expression analysis of the slightly decreased CD45+ cell subpopulation showed more dominant Igκ expression with κ/λ ratio of 7.30 (Additional file 1: Fig. S1C). Light chain expression analysis of the other 3 cases also showed slightly more dominant light chain expression, as described in Additional file 2: Table S1. We speculate that identifying a subpopulation of cells with slightly decreased CD45 expression may be useful for improving the accuracy of differential diagnoses between neoplastic B-cells (lymphoma cells) and the other cells (non-lymphoma cells), such as reactive inflammatory lymphoid changes.

CD10 is a proteolytic enzyme expressed on the surface of germinal center B-cells and lymphomas derived from these cells, that is, FL [23, 24]. A cut-off value of 20% is used to judge CD10 positivity [25]. In the present study, all FL cases were positive (> 20%) and MALT lymphoma cases were negative for CD10 (≤ 20%). These results are consistent with previous reports.

In conclusion, we could perform flow cytometric analysis with only 2 endoscopic biopsy specimens from the gastrointestinal tract. We consider that flow cytometric analysis of endoscopic biopsy specimens is feasible, and thus likely useful, for the diagnosis of gastrointestinal tract BCL in clinical settings.

## Limitations

- This was a prospective study involving a small number of patients conducted at a single center.
- Although the criteria for flow cytometric analysis of restricted light chain has been proposed for lymph nodes or bone marrow-derived B-cells, there have been no criteria for endoscopic biopsy specimens from patients with gastrointestinal tract BCL.

## Supplementary information

**Additional file 1: Fig S1.** Better selection of clonal B-cells by a subpopulation of cells with slightly decreased CD45 expression on Case 6. **A** Isolated cells (41.80%) (gate A) showed CD45 expression. **B** Light chain expression analysis of gate A showed dominant Igκ expression with κ/λ ratio of 3.69. **C** Analysis of subpopulation of cells with slightly decreased CD45 expression (gate B) revealed more dominant Igκ expression with κ/λ ratio of 7.30.

**Additional file 2: Table S1.** Comparison between a slightly decreased CD45+ and a CD45+ cell population. Light chain expression analysis showed more dominant light chain expression in a slightly decreased CD45+ cell population than in a CD45+ cell population.

## Data Availability

The datasets used and analysed during the current study are available from the corresponding author on reasonable request.

## Abbreviations

FCM: flow cytometry
BCL: B-cell lymphoma
FL: follicular lymphoma
