# Supplementary figures and images for: Feasibility of flow cytometric analysis of restricted light chain in endoscopic biopsy specimens from patients with gastrointestinal tract B cell lymphoma: a pilot study
Source: BMC Res Notes. 2019 Sep 11;12:571. doi: 10.1186/s13104-019-4578-4 (PMC6737588; doi:10.1186/s13104-019-4578-4)

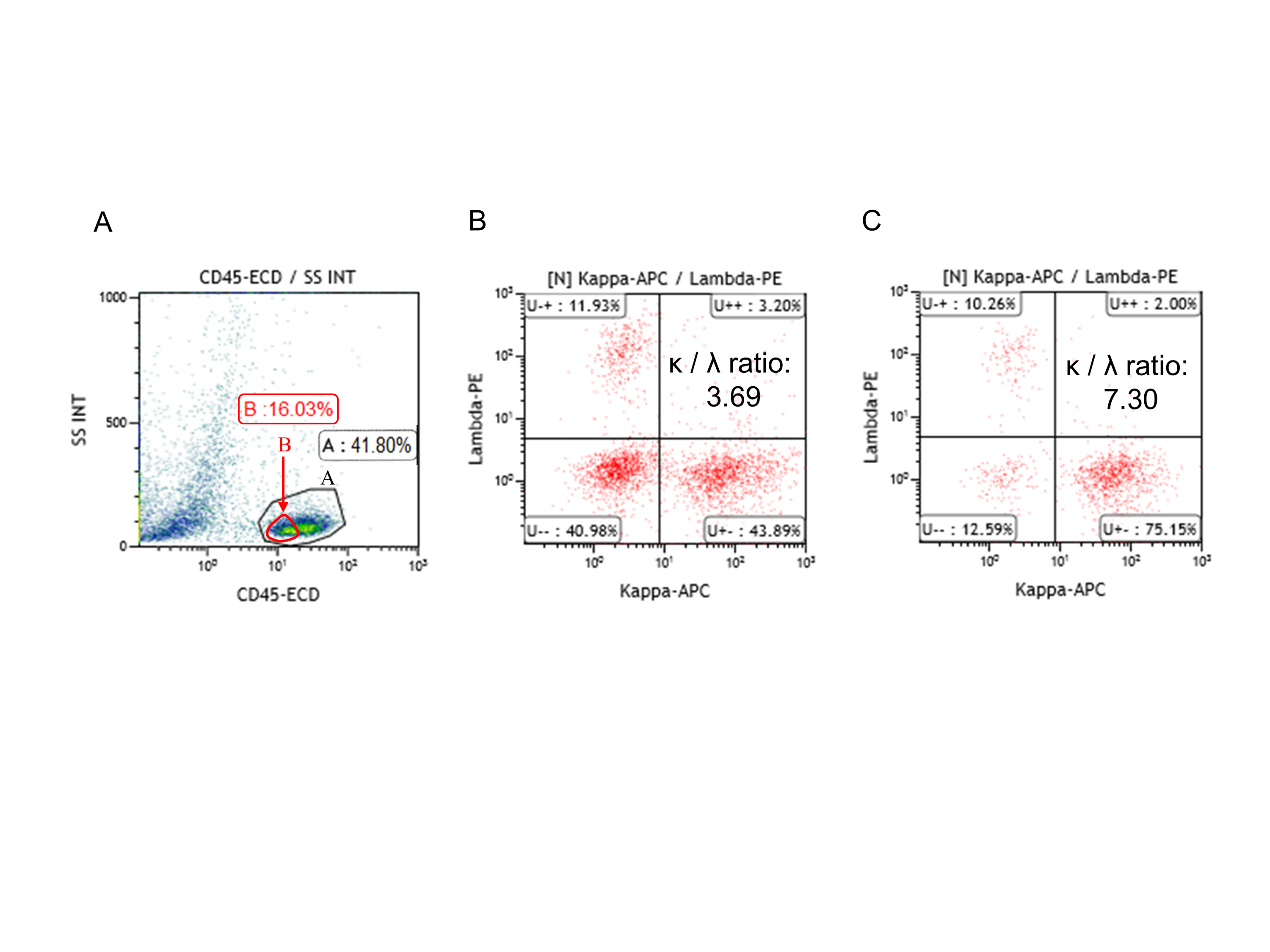

Supplement: Supplementary file 1 — Additional file 1: Fig S1. Better selection of clonal B-cells by a subpopulation of cells with slightly decreased CD45 expression on Case 6. A Isolated cells (41.80%) (gate A) showed CD45 expression. B Light chain expression analysis of gate A showed dominant Igκ expression with κ/λ ratio of 3.69. C Analysis of subpopulation of cells with slightly decreased CD45 expression (gate B) revealed more dominant Igκ expression with κ/λ ratio of 7.30. [file 13104_2019_4578_MOESM1_ESM.tif]
