# Supplementary material for: Feasibility of flow cytometric analysis of restricted light chain in endoscopic biopsy specimens from patients with gastrointestinal tract B cell lymphoma: a pilot study
Source: BMC Res Notes. 2019 Sep 11;12:571. doi: 10.1186/s13104-019-4578-4 (PMC6737588; doi:10.1186/s13104-019-4578-4)
Supplement: Supplementary file 2 — Additional file 2: Table S1. Comparison between a slightly decreased CD45+ and a CD45+ cell population. Light chain expression analysis showed more dominant light chain expression in a slightly decreased CD45+ cell population than in a CD45+ cell population. [file 13104_2019_4578_MOESM2_ESM.docx]

Table S1. Comparison between a slightly decreased CD45+ and a CD45+ cell population

| Case | Histologic diagnosis | Antigen expression (%)  in slightly decreased CD45+ cell population | | | Antigen expression (%)  in CD45+ cell population | | |
| --- | --- | --- | --- | --- | --- | --- | --- |
|  |  | Igκ | Igλ | κ / λ | Igκ | Igλ | κ / λ |
| 1 | MALT | 2.8 | 81.2 | 0.03 | 3.1 | 57.7 | 0.05 |
| 5 | MALT | 13.5 | 62.6 | 0.22 | 11.2 | 51.2 | 0.22 |
| 6 | MALT | 75.2 | 10.3 | 7.30 | 43.9 | 11.9 | 3.69 |
| 7 | FL | 45.7 | 1.6 | 28.56 | 36.7 | 1.5 | 24.47 |

MALT, MALT lymphoma; FL, follicular lymphoma;
